# A Patient With Novel *PPP1CB-ALK* Fusion Advanced NSCLC Achieved Long Survival From Alectinib: A Case Report

**DOI:** 10.1016/j.jtocrr.2026.100993

**Published:** 2026-03-26

**Authors:** Chang’e Jin, Chenhui Li, Ting Zhang, Jinfan Qiu, Min Guo, Zhengqiang He, Di Wu

**Affiliations:** Department of Pulmonary and Critical Care Medicine, Shenzhen Key Laboratory of Respiratory Diseases, Shenzhen Clinical Research Center for Respiratory Disease, Shenzhen Institute of Respiratory Diseases, Shenzhen People's Hospital (The First Affiliated Hospital, Southern University of Science and Technology; The Second Clinical Medical College, Jinan University), Shenzhen, Guangdong, People's Republic of China

**Keywords:** *PPP1CB*-ALK, Non–small cell lung cancer, Alectinib, Case report

## Abstract

ALK gene fusions are well-established oncogenic drivers that occur in approximately 5% of NSCLCs. The *EML4* gene is the most frequent fusion partner. With the advent of next-generation sequencing, numerous novel *ALK* fusions have been discovered; however, many have not been represented in pivotal clinical trials evaluating *ALK* tyrosine kinase inhibitors. We present a case of advanced NSCLC with a rare *PPP1CB-ALK* fusion. The patient achieved a durable response to alectinib, with a progression-free survival of nearly four years, and the patient continues to be progression free at the most recent follow-up. This case expands the spectrum of *ALK* fusion partners in NSCLC and contributes additional clinical evidence supporting the efficacy of alectinib therapy in rare *ALK* fusions.

## Introduction

In NSCLC, several *ALK* tyrosine kinase inhibitors (TKIs), including crizotinib, alectinib, and lorlatinib, have been approved for the treatment of advanced NSCLC with *ALK* fusions. With the advent of next-generation sequencing (NGS), more than 90 distinct *ALK* fusion partners have been identified, which exhibit variable clinical responses to ALK-TKIs.^1^ However, clinical data on rare *ALK* fusions in NSCLC remain scarce, hindering clear guidance on whether chemotherapy or ALK-TKIs should be prioritized. Notably, several reports have suggested that alectinib may constitute a viable treatment option for rare *ALK* fusions.^2^^,^^3^ Here, we present a case of advanced NSCLC with a *PPP1CA-ALK* fusion, a rare variant initially described in treatment-naive NSCLC, in which the patient achieved a progression-free survival (PFS) exceeding four years.

## Case Presentation

**Figure 1:**
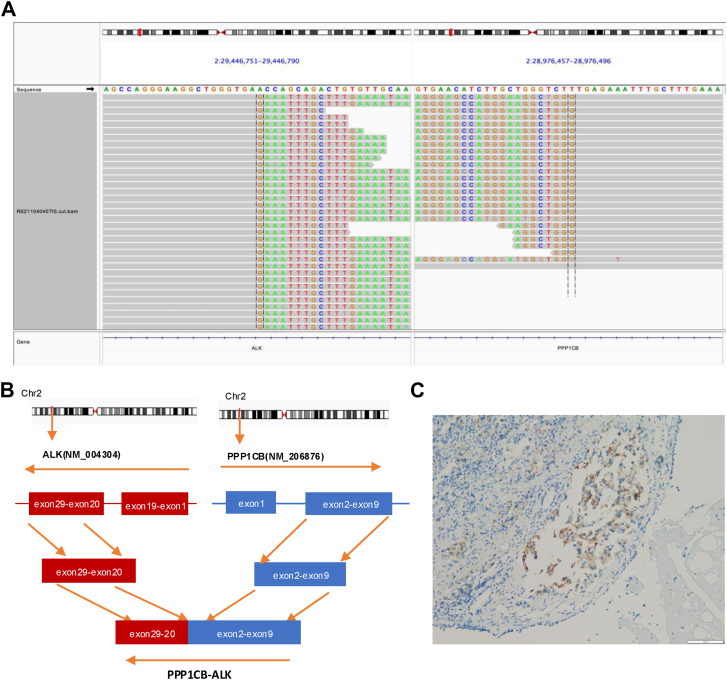
Identification of the *PPP1CB-ALK* fusion by next-generation sequencing and immunohistochemistry. (*A*) Integrative genomics viewer visualization of sequencing reads for *PPP1CB* and *ALK.* (*B*) Schematic representation of the *PPP1CB-ALK* fusion event. (*C*) Immunohistochemical staining revealing *ALK*-positive tumor cells.

**Figure 2:**
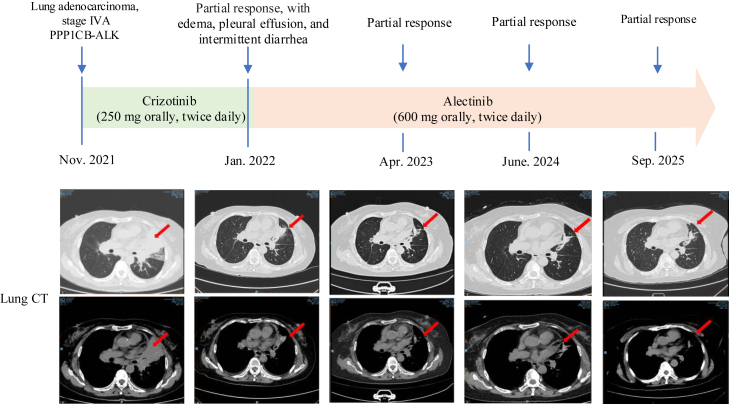
Clinical treatment history and imaging data of the patient.

A 71-year-old female patient with no history of smoking, an Eastern Cooperative Oncology Group performance status of 0, and no neurologic symptoms, such as headache or dizziness, presented to our hospital in October 2021 with a several-month history of progressive cough. Contrast-enhanced computed tomography of the chest revealed a centrally located carcinoma in the left lung, invading the left upper lobe artery and vein, accompanied by multiple metastatic lymph nodes in both hilar and mediastinal regions, including a suspected pleural metastasis on the left side. Abdominal contrast-enhanced computed tomography suggested possible adrenal metastasis, whereas contrast-enhanced brain magnetic resonance imaging revealed intracranial enhancing lesions with imaging characteristics consistent with metastatic disease. Bilateral bronchial mucosal biopsies were performed, and histopathologic examination confirmed lung adenocarcinoma. Immunohistochemical staining revealed CK7 (+), TTF-1 (+), Napsin A (+), P63 (−), CK5/6 (−), HER2 (1+), and PD-L1 (22C3, <1%) (Supplementary Fig. 1). Follow-up capture-based NGS was performed using a panel covering 520 cancer-related genes (Burning Rock Biotech, Guangzhou, China), and the NGS results revealed a *PPP1CB-ALK* fusion (Fig. 1*A* and *B*). Immunohistochemistry results further confirmed a weak positive *ALK* protein expression (Fig. 1*C*), which is a novel, and have not been previously reported in treatment-naive NSCLC. The patient initiated treatment with crizotinib (250 mg orally, twice daily) in November 2021. After one month of therapy, partial re-expansion of the collapsed left upper lobe and shrinkage of hilar and mediastinal lymph node lesions were observed. However, due to edema, pleural effusion, and intermittent diarrhea, crizotinib was discontinued and replaced with alectinib (600 mg orally, twice daily). Following the switch, the patient’s condition remained stable. Cytologic examination of pleural effusion obtained by thoracentesis revealed no malignant cells, and the pleural fluid gradually decreased. Subsequent follow-up imaging demonstrated continued tumor shrinkage, including gradual regression of the intracranial lesions on brain magnetic resonance imaging. At the most recent follow-up, the patient had achieved a PFS of 46 months. The treatment flowchart for this patient is depicted in Figure 2.

## Discussion

*PPP1CB-ALK* is a rare *ALK* fusion previously reported in isolated cases of infantile glioma and leiomyosarcoma,^4^^,^^5^ with no prior reports on treatment outcomes. Preclinical studies reveal that *PPP1CB-ALK* is oncogenic and sensitive to *ALK* inhibitors. One recent case described an *EGFR*-mutant high-grade neuroendocrine carcinoma of the lungs with concurrent *MRPL13-ALK* and *PPP1CB-ALK* fusions, treated with osimertinib plus crizotinib, but severe adverse effects led to discontinuation, and the patient died after one month.^6^ Thus, the clinical benefit of ALK-TKIs in *PPP1CB-ALK*–positive tumors remains unclear. Unlike canonical *EML4-ALK f*usions, which confer robust responses to ALK-TKIs, rare non–*EML4-ALK* fusions demonstrate variable but generally favorable responses. NGS has identified numerous rare *ALK* partners in NSCLC, including *KIF5B-ALK*, *HIP1-ALK*, and *STRN-ALK*. Many uncommon fusions respond to next-generation ALK inhibitors such as alectinib, though responses are heterogeneous and optimal strategies remain undefined.^7^^,^^8^ Our case represents the first patients with NSCLC with a *PPP1CB-ALK* fusion treated with alectinib, achieving a durable partial response and a prolonged PFS of 46 months, supporting the potential sensitivity of this rare fusion to modern ALK-TKIs. These findings emphasize the value of comprehensive molecular profiling and suggest that patients with rare *ALK* fusions may benefit from precision therapy.

## Conclusions

To our knowledge, this is the first reported case of a *PPP1CB-ALK* fusion in a patient with NSCLC treated with alectinib. The patient achieved a durable partial response, with a PFS of 46 months. Continued follow-up is ongoing to monitor disease progression. This case highlights the therapeutic potential of *PPP1CB-ALK* as an actionable target and provides valuable evidence for guiding treatment strategies in NSCLC with rare *ALK* fusions.

## CRediT Authorship Contribution Statement

**Chang’e Jin:** Conceptualization, Methodology, Resources, Writing - original draft, Writing - review & editing.

**Chenhui Li:** Methodology, Validation, Formal analysis, Data curation, Writing - original draft, Writing - review & editing.

**Ting Zhang:** Writing - original draft, Writing - review & editing.

**Jinfan Qiu:** Writing - review & editing.

**Min Guo:** Writing - review & editing.

**Zhengqiang He:** Resources, Writing - review & editing.

**Di Wu:** Conceptualization, Methodology, Resources, Supervision, Writing - original draft, Writing - review & editing.

## Disclosure

The authors declare no conflict of interest.
