# Supplementary figures and images for: A Patient With Novel PPP1CB-ALK Fusion Advanced NSCLC Achieved Long Survival From Alectinib: A Case Report
Source: JTO Clin Res Rep. 2026 Mar 26;7(6):100993. doi: 10.1016/j.jtocrr.2026.100993 (PMC13181242; doi:10.1016/j.jtocrr.2026.100993)

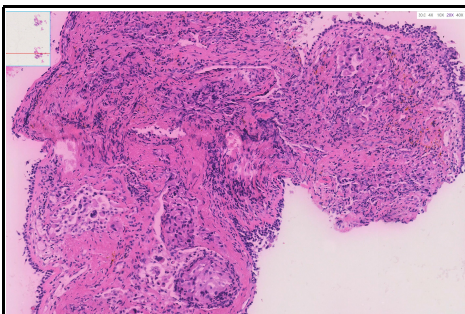

**H&E**

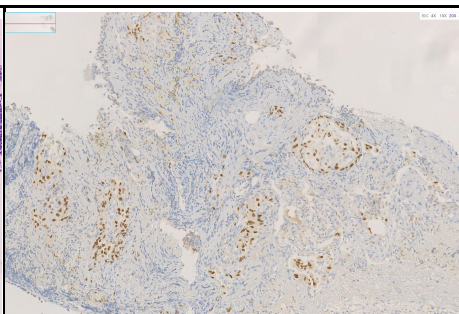

**TTF-1 Positive**

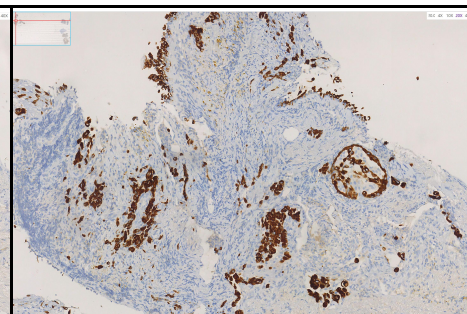

**CK7 Positive**

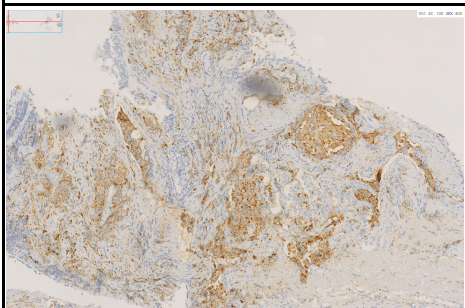

**Napsin A Positive**

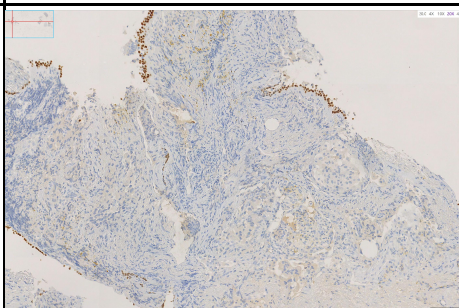

**P63 Negative**

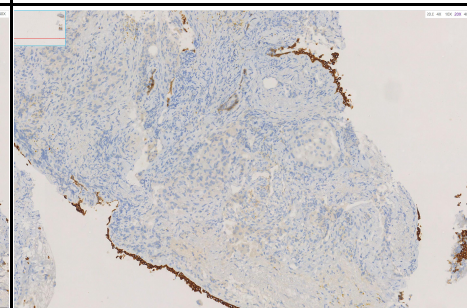

**CK5/6 Negative**

Supplement: Figure S1 [file mmc1.pdf]
